# Temporal dynamics of isolation calls emitted by pups in environmental and genetic mouse models of autism spectrum disorder

**DOI:** 10.3389/fnins.2023.1274039

**Published:** 2023-10-23

**Authors:** Ayelet Gal, Eynav Raykin, Shaked Giladi, Dror Lederman, Ora Kofman, Hava M. Golan

**Affiliations:** ^1^Department of Physiology and Cell Biology, Ben-Gurion University of the Negev, Beer Sheva, Israel; ^2^Psychology Department, Ben-Gurion University of the Negev, Beer Sheva, Israel; ^3^Faculty of Engineering, Holon Institute of Technology Holon, Holon, Israel; ^4^National Center for Autism Research, Ben-Gurion University of the Negev, Beer Sheva, Israel

**Keywords:** autism, MTHFR, chlorpyrifos, ultrasonic vocalization, communication, neonate

## Abstract

**Introduction:**

Environmental and genetic factors contribute to the increased risk for neurodevelopmental disorders, including deficits in the development of social communication. In the mouse, ultrasonic vocalizations emitted by the pup stimulate maternal retrieval and potentiate maternal care. Therefore, isolation induced ultrasonic vocalization emitted by pups provides a means to evaluate deficits in communication during early development, before other ways of communication are apparent. Previous studies in our labs showed that gestational exposure to the pesticide chlorpyrifos (CPF) and the Methylenetetrahydrofolate (Mthfr)-knock-out mice are associated with impaired social preference, restricted or repetitive behavior and altered spectral properties of pups’ ultrasonic vocalization. In this study, we explore the temporal dynamics of pups’ vocalization in these Autism spectrum disorder (ASD) models.

**Methods:**

We utilized the maternal potentiation protocol and analyzed the time course of pup vocalizations following isolation from the nest. Two models of ASD were studied: gestational exposure to the pesticide CPF and the Mthfr-knock-out mice.

**Results:**

Vocalization emitted by pups of both ASD models were dynamically modified in quantity and spectral structure within each session and between the two isolation sessions. The first isolation session was characterized by a buildup of call quantity and significant effects of USV spectral structure variables, and the second isolation session was characterized by enhanced calls and vocalization time, but minute effect on USV properties. Moreover, in both models we described an increased usage of harmonic calls with time during the isolation sessions.

**Discussion:**

Communication between two or more individuals requires an interplay between the two sides and depends on the response and the time since the stimulus. As such, the presence of dynamic changes in vocalization structure in the control pups, and the alteration observed in the pups of the ASD models, suggest impaired regulation of vocalization associated with the environmental and genetic factors. Last, we propose that temporal dynamics of ultrasonic vocalization communication should be considered in future analysis in rodent models of ASD to maximize the sensitivity of the study of vocalizations.

## Introduction

With the increasing effort to find early markers for later abnormal neurodevelopment in human babies and rodent models of neurodevelopmental disorders, attention was given to ultrasonic vocalization (USV) emitted by rodent pups as a means of attracting maternal attention. Mouse USVs (>30 KHz) are produced by air streams mediated by laryngeal neurons at the level of the midbrain and lower brainstem (Tschida et al., 2019; Riede et al., 2022; Wei et al., 2022). In addition, forebrain premotor pathways enable production of USVs in response to distress and touch, modified by environmental and social contexts (Fitch et al., 2010). The number of calls and duration of adult rodent USVs are modified by social and emotional factors (Branchi et al., 1998; Mai et al., 2023). Longer duration and lower frequency USVs are more abundant under aversive conditions, whereas short duration, higher frequency USVs are emitted in positive affective states (Boulanger-Bertolus et al., 2017). Social contexts, such as the presence of an opposite sex conspecific, or the presence of mice from another population also modify the emission of USVs (Wang et al., 2008; von Merten et al., 2014). Anxiety-provoking situations (Wöhr and Schwarting, 2013) and USV production are modified by anxiolytic drugs (Hodgson et al., 2008). The importance of social cues in modifying adult rodent USVs led to the exploration of USV production in mouse models of autism spectrum disorder (ASD), a disorder defined by deficits in social communication and affiliation (Crawley, 2007).

USV production in rodent pups is not dependent on imitation or learning (Fischer and Hammerschmidt, 2011). USV are produced even before ear opening (Grimsley et al., 2011) and the properties of USVs of deafened mice are not significantly different from those of intact mouse pups (Mahrt et al., 2013). Initially, USVs were thought to be a byproduct of abdominal compression in response to falling, cold temperature or rough handling (Shair et al., 2003; Portfors, 2007). However, ample evidence shows that pup USVs are modulated by the presence of adults, as shown in reduction in the presence of an unfamiliar male and an increase in the presence of a familiar adult of either sex (Shair, 2014). Adult male and female mice, including sexually naïve adults, exhibit phonotaxis, movement toward the source of pup USVs. The latency to the dam’s response depends on her hormonal and behavioral state (Ehret, 2005; Portfors, 2007). While USVs serve as a behavioral sign that stimulates approach, continued production of USVs is not required to complete the retrieval process by the dam. The dam will complete pup retrieval based on other sensory cues once the isolated pup has been located (Ehret, 2005). USVs potentiate maternal care (D'Amato et al., 2005; Shair, 2014; Tasaka et al., 2020), and mating in adults (Portfors, 2007). In conclusion, although USV production is present in deaf, as well as hearing pups, the social aspect of USV production generated research on this behavior as a potential marker of ASD like behavior (Scattoni et al., 2009).

Maternal potentiation of the rate of pup USV production occurs in an isolation period that follows a previous isolation and reunion with the dam (Shair et al., 1997, 2003). The influence on pup USV production is based on the pup’s memory of the first isolation session and the “reward” to its calls, and Shair deemed it to be, “*a marker for filial attachment”* (Shair, 2014). Moreover, USV emission after maternal potentiation depends on the quality of maternal care during reunion with the pup (D'Amato et al., 2005). Inbred mouse models of ASD are based on purported genetics (e.g., fragile X FMR1 mice) and environmental risk factors. Other mouse models are based on a documented behavioral profile that mimics core ASD features, such as the BTBR mice (Scattoni et al., 2013; Perets et al., 2018) or PON1-mice (Kofman et al., 2020). Despite similarity in the core markers of ASD behavior, such as reduced social preference and increased behavioral perseveration, the various murine ASD model pups show a wide range and inconsistent patterns of USV reactions to isolation from their litter. This can be seen with respect to USV number, duration and spectral structure. For example, the vasopressin receptor type V1b Avpr1b null pups did not show an overall difference in the number of USVs, but failed to show maternal potentiation of USVs upon retesting at age P9 (Scattoni et al., 2008). BTBR mice emit more isolation USVs than WT mice (Scattoni et al., 2013) but Engrailed 2 (Brielmaier et al., 2012), and the FMR1 mutation pups (Nolan et al., 2017, 2020) did not differ from their respective wild type control groups when the abundance of isolation USVs was assessed. Different patterns of USV production have been reported in the background strains, C57Bl/6 and Balb/C, the mice used in this study, with B6 mice showing higher frequencies and shorter durations in adolescents (Panksepp et al., 2007) and in pups (Shekel et al., 2021). Because the goal of our research was to examine both qualitative and quantitative features of USVs in the maternal potentiation paradigm, we conducted parallel analyses on each ASD murine model compared to their respective control groups, in order to define the common variables related to aberrant USV production.

To assess the common features modified by pups of an environmental and a genetic model of ASD (Lan et al., 2017; Sadigurschi and Golan, 2018; Lan et al., 2019; Sadigurschi et al., 2023), we previously compared the features of USVs emitted by mouse pups following gestational exposure to the organophosphate chlorpyrifos (CPF), and in a genetic model of Methylenetetrahydrofolate (Mthfr) haploinsufficiency (Mthfr, Entrez Gene ID: 4524). As described in previous publications, both mouse models were confirmed to have delayed developmental sensorimotor reflexes and deficits in the social preference test that is widely used to assess ASD in rodents (Moy et al., 2009). Our collaborative research comparing USV features in these diverse ASD models, used unbiased clustering of USV spectral properties. We found that the start and end frequency and duration of USVs were most sensitive to the genetic and environmental factors and showed the highest change in several USV cluster types (Shekel et al., 2021). The goal of the current study is to conduct an analysis of the temporal dynamics of pups’ vocalizations in the maternal isolation paradigm on these two ASD models. We used the maternal potentiation protocol to investigate whether there is a characteristic change in the timing and spectral properties of the call (a) during the first isolation and (b) in response to reunion with their dam in the second separation (Shair et al., 2003).

Pup USVs show an age-dependent reduction in call length and bandwidth within the first 2 postnatal weeks (Noirot and Pye, 1969). We analyzed calls from postnatal day (P) 8, similar to other ASD vocalization studies (Branchi et al., 1998), as this day falls within the peak USV production period of preweanling mice (Elwood and Keeling, 1982). We used the “classical” USV categories as presented in early studies (Scattoni et al., 2008), and grouped these USV into 3 categories of USV complexity. USV calls emitted by pups of both models of ASD, were dynamically modified in quantity and spectral structure within each session and between the isolation sessions, with the first isolation session characterized with a buildup of call quantity and significant effect on USV acoustic structure, and the second isolation session characterized by enhanced calls and voice time, but minute effect on USV features.

## Methods

### Experimental design

Environmental model mice [C57Bl6J (B6)]: Dams and sires for breeding were purchased from Envigo, Israel. Dams were divided into 3 treatment groups for organophosphate exposure (CPF) during gestation: Vehicle (VEH, corn oil, *n* = 7), 2.5 (CPF-L, *n* = 10), or 5 mg/kg CPF (CPF-H, *n* = 9). Mthfr mice: Mice on a Balb/cAnNCrlBR background were studied (Chen et al., 2001) to assess the effect of maternal Mthfr+/− genotype vs. offspring genotype. Mthfr+/+ (wild-type [Wt]) and Mthfr+/− (heterozygote [Het]) female mice were mated with Wt males to create three groups defined by genotype and maternal genotype as follows: Wt offspring from Wt mothers (Wt:Wt, *n* = 4), Wt offspring from Mthfr+/− mothers (Het:Wt, *n* = 13) and Mthfr+/− offspring from Mthfr+/− mothers (Het:Het, *n* = 8). Mthfr−/− mice are not viable.

An illustration of the study design and description of experimental groups can be seen in Supplementary Figure 1A.

The mouse colonies were maintained on a 12:12 h light/dark schedule, temperature 21°C–23°C with *ad libitum* food and water. All procedures were performed according to the guidelines of the Israeli Council on Animal Care and approved by the Animal Care and Use Committee of Ben-Gurion University of the Negev, accredited by the Association for Assessment and Accreditation of Laboratory Animal Care (protocols IL-16-07-14 and IL-66-11-13).

### Prenatal exposure

Chlorpyrifos (CPF, 99.5% purity, Chem Service, Inc.) suspended in corn oil (Willi Food, Yavneh, Israel) or vehicle control was administered by gavage to B6 pregnant females daily from gestation day (GND) 12 to 15 in a volume of 0.1 mL/10 g body weight using a 22-gauge stainless steel feeding tube (Solomon Instech, Inc.). The dams were observed after gavage and showed no signs of cholinergic toxicity (Ricceri et al., 2007; Lan et al., 2017).

### Genotyping

Mthfr and Wt mice were genotyped using polymerase chain reaction, as previously described (Chen et al., 2001).

### Ultrasonic vocalization

#### Acoustic recording

Ultrasonic signals were recorded using Avisoft bioacoustics (Berlin, Germany) system including: Ultrasoundgate 116 Hm with The ultrasound microphone CM16/CMPA, using The Avisoft recorder 4.2.17—Bioacoustics recording software. Recordings were set at a sampling frequency of 250 kHz in a trigger mode using a threshold of 0.5% of the signal’s energy in the range of 10–250 kHz.

CPF model: One male from each litter, marked by snipping the end of the tails on P1, was randomly chosen. Mthfr model: 2–3 pups per litter were recorded and the sex and genotype were defined when pups reached P30. To enable comparison between the environmental and genetic model all pups were recorded on postnatal day 8. In total the numbers of mice analyzed for the current study were: CPF 26 male. Female mice were not analyzed in this study as they had not previously shown a deficit in social preference or conditioned social place preference (Lan et al., 2019). Mthfr, 9 male and 16 female.

Each pup was separated from the litter and placed in a transparent plastic cup (11 cm high and 10 cm diameter) located on a warm pad. The microphone was placed 10 cm above the pup. After a 10-min isolation session (S1) the pup was placed back in the home cage with the litter for 20 min and separated for a second recording session of 10 min (S2). The recording area was cleaned with ethanol (70%) between pups.

#### USV analysis

In order to analyze USV calls, we developed a designated algorithm based on spectrogram analysis. Briefly, the USV calls are divided into short segments of 0.5 s each. For each segment, the Fourier transform is calculated. Then, the energy of the signal for each frequency band is calculated and a pre-determined threshold is applied. The algorithm was implemented in Matlab and used to segment the USV calls, i.e., determine the beginning and end of each voice segment. Based on the segmentation results, the time and USV type was classified, by a researcher blind to the group identity, based on the classification suggested by Scattoni et al. (2008). The following variables were used to compare USV syllables between groups: Start Frequency, defined as mean frequency at start of the syllable, End Frequency, defined as mean frequency at the end of the syllable, Duration (the time difference between start and end of the call) and inter call interval (ICI, the time difference between the end of a call and the start of the following call), inter call intervals larger than 0.5 s were defined as inter burst intervals. Calls that were interrupted by noise or other recording problems were included only for the analysis of call quantity and call type but were omitted from the analysis of calls properties (duration, frequencies, and ICI).

#### Call complexity level

**Figure 1:**
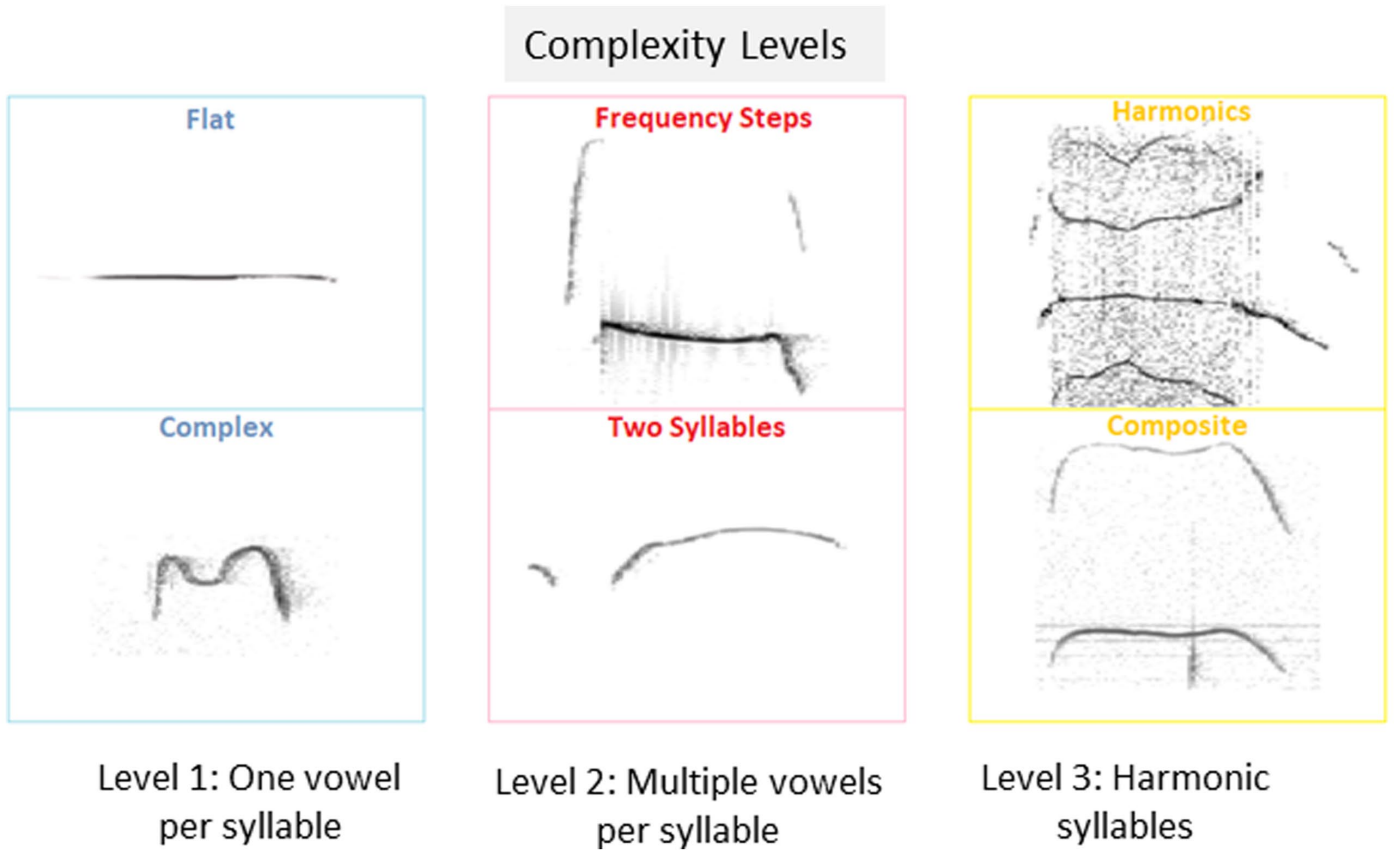
Isolation induced USV classification and proportions of pups emitting isolation induced USVs. Examples of isolation induced USV calls included in each of the 3 categories of complexity level: Level 1: USV with a single vowel, Level 2, USV with multiple vowels per syllable, and Level 3, USV with harmonic syllables used by pups in the current study.

Calls of the 10 types suggested before (Branchi et al., 1998; Scattoni et al., 2008) were classified as belonging one of 3 categories of complexity, as follows: Level 1 includes calls with a single vowel, i.e., flat, upward, downward, chevron, short and complex; Level 2 includes calls with multiple vowels per syllable, i.e., frequency steps and two syllables, and Level 3 includes harmonic calls, i.e., composite and harmonic, as shown in Figure 1.

### Statistical analysis

In order to capture the effect of the duration of the isolation, we sampled all the calls emitted by the pups during the first and sixth minutes of each of the maternal isolation sessions: 2141 calls from the CPF model and 3,175 calls from the Mthfr model. Unlike previous studies, that sampled a short period of calls continuously (Scattoni et al., 2009; Brielmaier et al., 2012; Nolan et al., 2020), this sampling method enabled us to investigate whether the call complexity was affected differentially in the treatment groups as the isolation proceeded over 10 min. Statistical analyses were performed by SPSS26 software (IBM) to test the effect of the fixed factors; experimental group, time (session and time within the session), and sex for the Mthfr mice. Analysis of parametric variables was performed by univariate (general linear model) test. Syllable usage among sessions and groups was compared using Chi-square test.

## Results

### Temporal changes in the quantity and type of calls emitted by male pups of environmental and genetic ASD models

Emission of USV calls by pups is enhanced by pup separation from the nest, and further enhancement was reported in the second session, following reunion with the mother and littermates. This basic form of communication between the pup and the mother is modified in genetic and environmental ASD models (Shekel et al., 2021).

Upon separation from the litter, about half of the pups in the control and experimental groups remained silent. Following reunion with the litter, in the second separation session (S2) the majority of pups emitted USV calls. Thus, a significant increase in the percent of vocal pups was observed in all groups (150%, 114%, 180% vehicle, CPF-L and CPF-H, respectively; see Supplementary Table 1).

**Figure 2:**
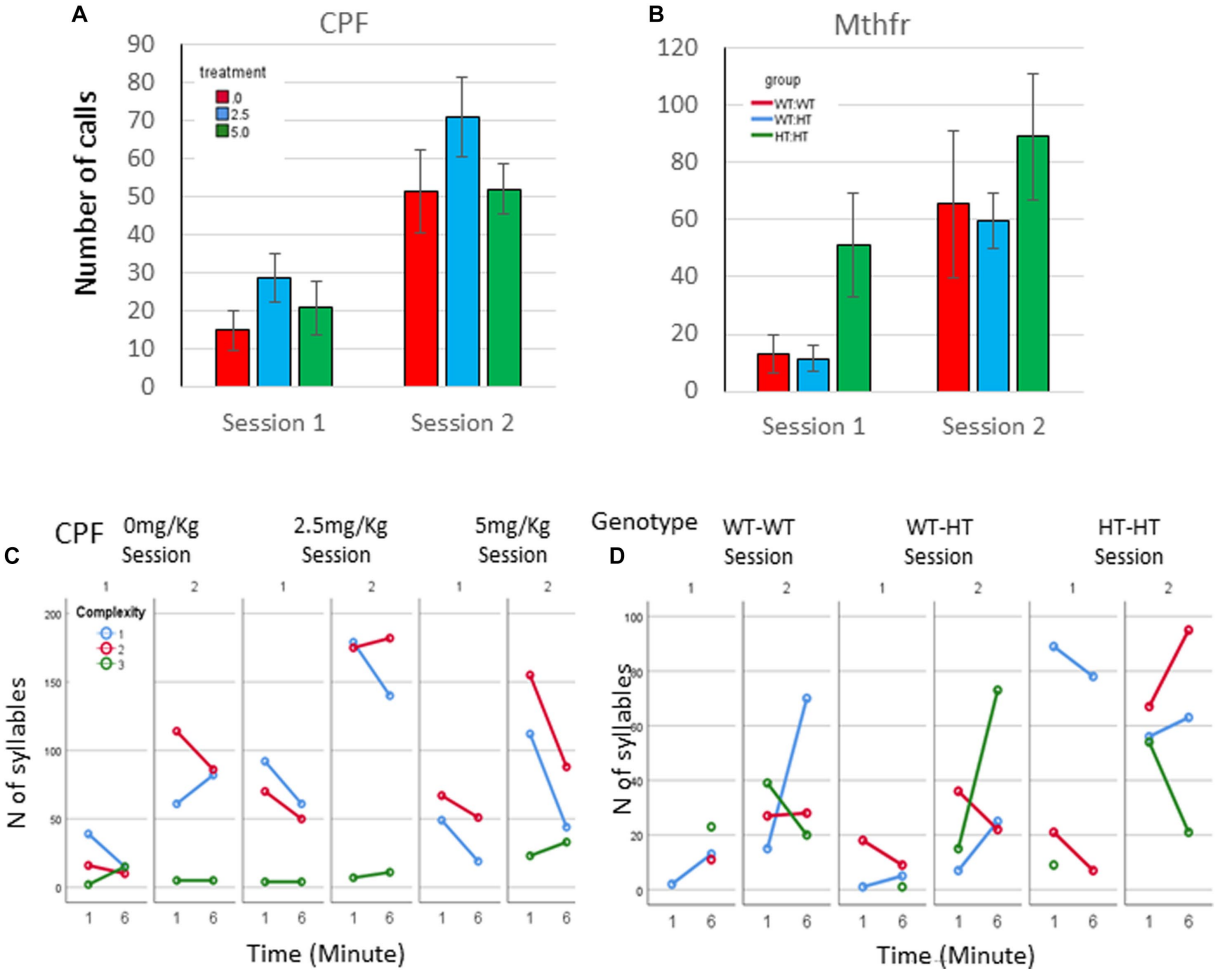
CPF treatment and Mthfr+/− genotype alters isolation induced call quantities, maternal potentiation and call complexity. The average number of USV syllables per pup in session 1 and 2. **(A)** CPF model, and **(B)** Mthfr model. Mean +/− SEM, N = CPF: 16 (Oil: 7, CPF-L: 10, CPF-H-9), Mthfr—Male: 9 (Wt:Wt-2, Het:Wt-3, Het:Het-4). **(C,D)** The number of USV syllables emitted by pups at the 1st and 6th minute of each isolation session, by complexity level. **(C)** CPF model, and **(D)** Mthfr model. Missing points or lines indicate that at that particular segment of time no calls of that type were made.

In addition to the increased number of vocal pups during S2, the number of calls emitted by pups was elevated. An overall potentiation on S2 (*F*_5,92_ = 14.54, *p* < 0.0001, Figure 2A), compared to number of calls emitted in S1 was observed in the CPF model. In the control group, there was a trend to an increase in the number of calls in S2 (306% and 395% of S1, for 1st and 6th minutes, *p* = 0.07). Potentiation of the number of calls was observed in the CPF-L (217, 293% *p* = 0.002), and CPF-H (252, 245%, *p* = 0.046) groups, as shown in Figure 2A and Supplementary Table 1.

Similar trends of potentiation were observed in male pups of the Mthfr model, induced by maternal and offspring genotypes, but did not reach significance (Figure 2B; Supplementary Table 1).

To further characterize the calls emitted during the test, we analyzed the type of calls emitted by pups, based on the 10 categories suggested previously (Scattoni et al., 2008), and further grouped these categories into 3 complexity levels (1–3), as shown in Figure 1 (see also Methods).

Mice in the CPF model present very low usage of level 3 calls. The vehicle group emitted mostly level 1 calls during S1 and enhanced the use of Level 1 and 2 calls in S2. In addition, CPF-L, emitted a higher number of Level 1 and 2 calls compared to control in S1, and further increased emission of these call types during S2. [Effect of treatment for the use of Level 1 *F*_11,92_ = 3.6, *p* = 0.031, effect of session (level 1) *F*_11,92_11.854, *p* < 0.001; (level 2) *F*_11,92_ = 11.524, *p* = 0.001]. CPF-H pup emitted mostly Level 1 calls during S1 and expanded their repertoire of calls in S2 to all calls type (level 1–3). The changes in the use of level 3 calls are indicated also by an interaction between treatment and session *F*_11,92_ = 3.337, *p* = 0.038, Figure 2C. To ease evaluation of the relative changes induced by CPF exposure, call quantity by complexity was expressed as percentage of the calls emitted during the first minute of the test (see Supplementary Figure 2A).

In the Mthfr model, the enhancement in the number of calls included an increase in the number of calls of all complexity levels, with a significant effect of session on the number of calls of levels 2 and level 3 (*F*_5,30_ = 5.125, *p* = 0.031 and *F*_5,30_ = 11.689, *p* = 0.002, for calls of level 2 and 3, respectively; Figure 2D; Supplementary Figure 2B).

As a whole, pups from both models, control and experimental groups present an increase in the number of vocal pups that emitted USVs and in the number of USVs emitted by the pups in the second isolation S2 that followed the reunion with the litter. In addition to the number of calls, the usage of calls at different complexity levels was modulated with time (min and session) as shown by the interactions between min * session * treatment, *F*_3,2,110=_14.929, *p* = 0.000, and the interaction between min * session * genotype, *F*_4,926_ = 8.710, *p* = 0.000. Pups with the highest prenatal organophosphate exposure or genetic load increased the usage of level 3 calls, during S2, compared to S1 (Figure 2).

To compare the effect of organophosphate and genetic exposure, to the dynamic changes in the control groups, the effect of treatment is shown in Supplementary Table 1 as the percent change in the number of calls at the 1st and 6th minute of each session compared to the control group.

### USV calls features are dynamically modulated in environmental and genetic models of ASD

**Figure 3:**
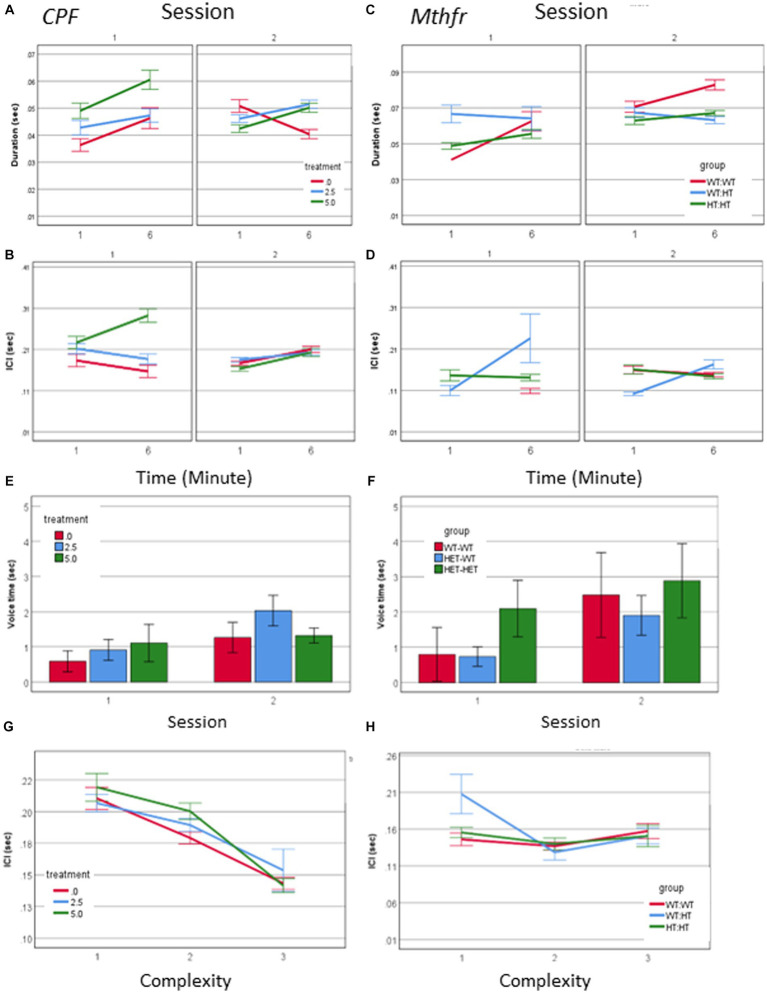
CPF treatment and Mthfr+/− genotype alter the temporal properties of male pup vocalization. Duration of the USV calls and ICI in the CPF model **(A,B)**, and in the Mthfr model **(C,D)**, Mean +/− SE,N = CPF: 2,113 (Oil: 463, CPF-L: 995, CPF-H-655), Mthfr—925 (Wt:Wt-157, Het:Wt-212, Het:Het-560). Mean voice duration in the CPF model and the Mthfr model **(E,F)**. The relation between ICI and USV complexity level in the CPF model and the Mthfr model **(G,H)**. Missing points or lines indicate that at that particular segment of time no calls of that type were made. Mean +/− SE, N = CPF: 16 (Oil: 7, CPF-L: 10, CPF-H-9), Mthfr—Male: 9 (Wt:Wt-2, Het:Wt-3, Het:Het-4).

Call duration was modified by all independent factors and showed an interaction between treatment * minute * session as presented in Figure 3A (*F*_2,2054_ = 4.098, *p* = 0.017). When evaluating the vehicle group for the effect of the time (min and sessions) on call duration, as a baseline for the study of CPF treatment effects there was an interaction between minute * session (*F*_2,448_ = 11.228, *p* < 0.001). Thus, all comparisons between the groups are done on a dynamic background of call duration in the vehicle group. For example, during S1, the Vehicle group emitted longer calls in the 6th min compared to the 1st min. This interaction is not present in the CPF groups, in which there are consistent main effects of both min and session, with no interaction between these variables (CPF-H effect of minute *F*_2,973_ = 13.653, *p* < 0.001, and effect of session *F*_2,639_ = 17.524, *p* < 0.001). Despite the dynamic changes in the duration of calls emitted by pups in the vehicle group, calls emitted by the CPF-H pups were as long as, or longer than calls in the vehicle group at each time tested (effect of treatment *F*_2,2054_ = 6.542, *p* = 0.001). The inter call interval (ICI) also dynamically changed in the vehicle group with an interaction between minute * session (*F*_2,448_ = 7.798, *p* = 0.006). In the CPF exposed mice there was an interaction between treatment * minute * session (*F*_2,1,594_ = 4.123, *p* = 0.016) on the ICI. In contrast to the vehicle group, in which the ICI was reduced with time during S1, in the CPF-H pups the ICI was initially longer and lengthened further as S1 progressed. In S2 a similar effect of time was observed in all groups with longer ICI in the 6th min compared to 1st min (*F*_2,1,594_ = 26.490, *p* = 0.000), as shown in Figures 3A,B.

Call duration in male pups of the Mthfr model presented an interaction between Mthfr genotype and session (*F*_1,847_ = 7.009, *p* = 0.008), while in the Wt offspring call duration was stable in both sessions (Figure 3B). In the Mtfr+/− pups the duration was longer in S2 and was affected by each of these variables; Mthfr genotype and session (Shorter call duration as a whole, and longer duration in S2, in the Mthfr+/− pups compared to Wt pups, *F*_df1,847_ = 7.509, *p* = 0.006, and *F*_1,847_ = 9.897, *p* = 0.002, respectively; Figure 3C). In addition, in these pups, there was an interaction between maternal Mthfr genotype * session (*F*_1,847_ = 7.045, *p* = 0.008) on ICI. Maternal and offspring Mthfr genotypes interacted also with the time during each session (minute; *F*_1,847_ = 11.793, *p* = 0.001 and *F*_1,847_ = 23.958, *p* < 0.001, respectively), while in pups of Wt dams ICI was stable during the session. In pups of Mthfr+/− dams an increase in ICI was observed within each session (Figure 3D), data are presented also in Supplementary Table 2.

To explore the relation between the number of calls and call duration we computed the total voice duration within the tested minutes (1 and 6). The voice duration was longer in S2 compared to S1 (effect of session *F*_2,70_ = 4.059, *p* = 0.048), and was not affected by the treatment (Figure 3E). Longer voice time in S2 compared to S1 was found also in pups of the Mthfr model (effect of session *F*_2,70_ = 6.270, *p* = 0.018) which was not modified by Mthfr genotype in the male pups (Figure 3F).

The temporal dynamics of call sequences was evaluated also by the relation between call type and the following ICI. We found a significant negative correlation between call complexity level and the following ICI duration (Pearson R = −0.142, *p* < 0.001), with longer ICIs following simple calls, as shown in Figure 3G. These relations were not affected by prenatal organophosphate CPF.Negative ICI vs. complexity relations were not observed in any pups of the Mthfr model, as can be seen in Figure 3H, in which only calls emitted by the Het-Wt pups preserved this tendency between call ICI and complexity.

**Figure 4:**
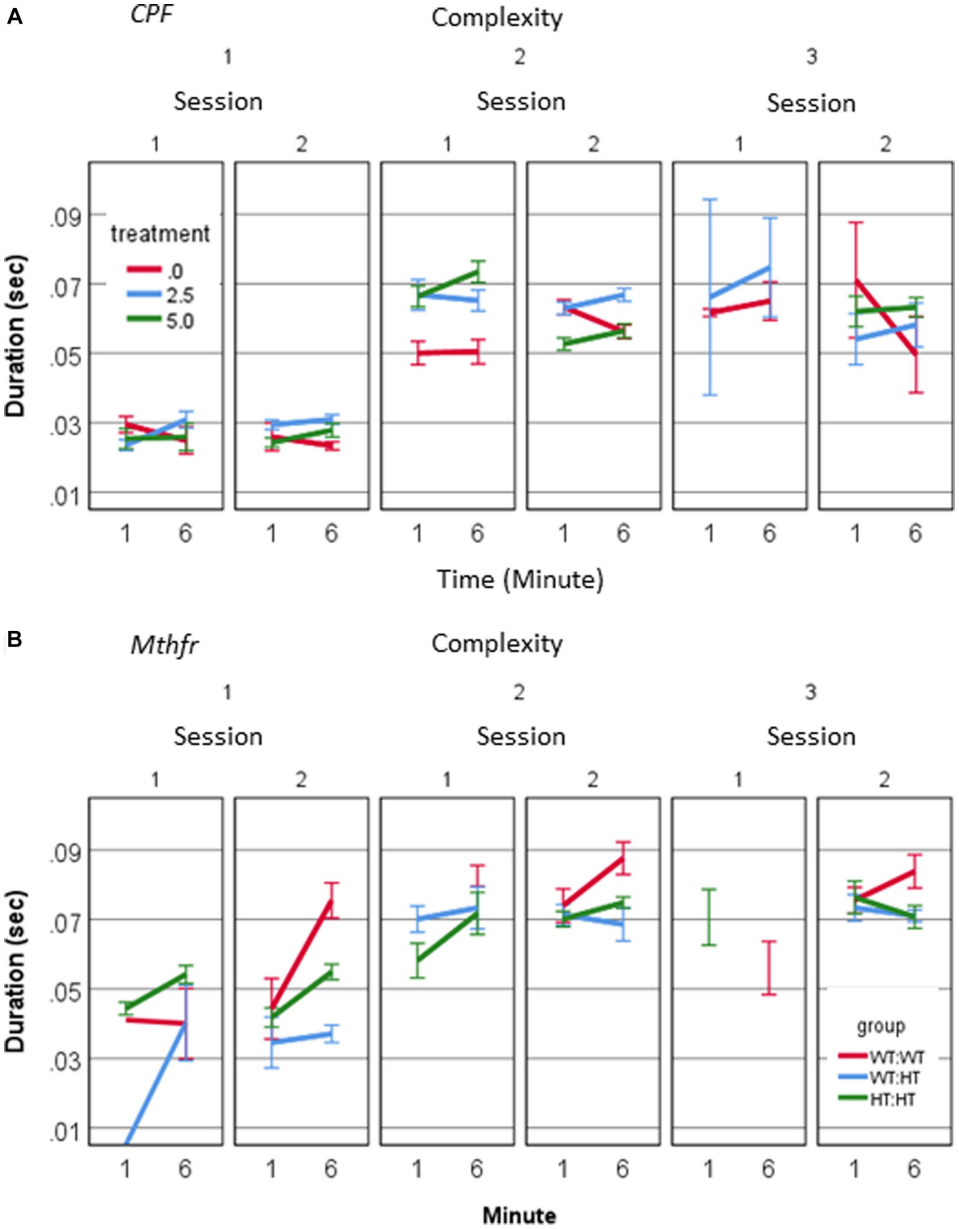
CPF treatment and Mthfr+/− genotype alter the duration of isolation induced calls of male pup by complexity levels. **(A)** Mean duration of USV calls by complexity level, in the CPF model **(A)** and the Mthfr model **(B)**. Mean +/− SE, N = CPF: 2113 (Oil: 463, CPF-L: 995, CPF-H-655), Mthfr—925 (Wt:Wt-157, Ht:Wt-212, Het:Het-560).

A source of longer vocalization can be related to the usage of short vs. long calls, or change in the duration of particular call type. A positive relation between call duration and their complexity level (Pearson R = 0.580, *p* < 0.001) was found. To explore the possibility that a change in the duration appears in particular call types, we examined the interaction between treatment, call complexity level, minute and session. A significant effect of session on the duration of level 2 calls in the vehicle group (*F*_1,192_ = 7.235, *p* = 0.008) and CPF-H (*F*_1,284_ = 41.384, *p* = 0.000), Figure 4A was found. In summary, CPF induced an increase in pups’ voice time, which may be due to both more level 2 and 3 calls (Figure 2A) and to the longer duration of level 2 calls (Figure 4A).

In the male pups of the Mthfr model, call duration increased with complexity (Pearson R = 0.452, *p* < 0.0001). In addition, a significant effect of offspring Mthfr genotype on call duration was observed for level 1 calls, which differed between sessions depending on offspring Mthfr genotype (genotype * session * min, *F*_1,296_ = 5.314, *p* = 0.022). The effect of offspring genotype on the duration was significant for Level 1 calls (*F*_1,296_ = 6.1, *p* = 0.014), but not in the more complex calls (Levels 2 and 3; Figure 4B).

The rhythm of the vocalizations was significantly affected by both maternal and offspring Mthfr genotype. The change in rhythm was expressed as a significant effect on the ICI following Level 2 and 3 calls (Level 2: genotype * session *F*_1,267_ = 6.677, *p* = 0.01, genotype * min; *F*_1,267_ = 10.962, *p* = 0.001, Maternal genotype * min *F*_1,267_ = 4.688, *p* = 0.031; Level 3: Maternal genotype *F*_1,189_ = 4.755, *p* = 0.030). Thus, in the Mthfr model, rhythmicity of USV production was altered either by call duration or intervals between calls, depending on the level of call complexity.

### Spectral properties of USV calls undergo dynamic modulation during isolation sessions

**Figure 5:**
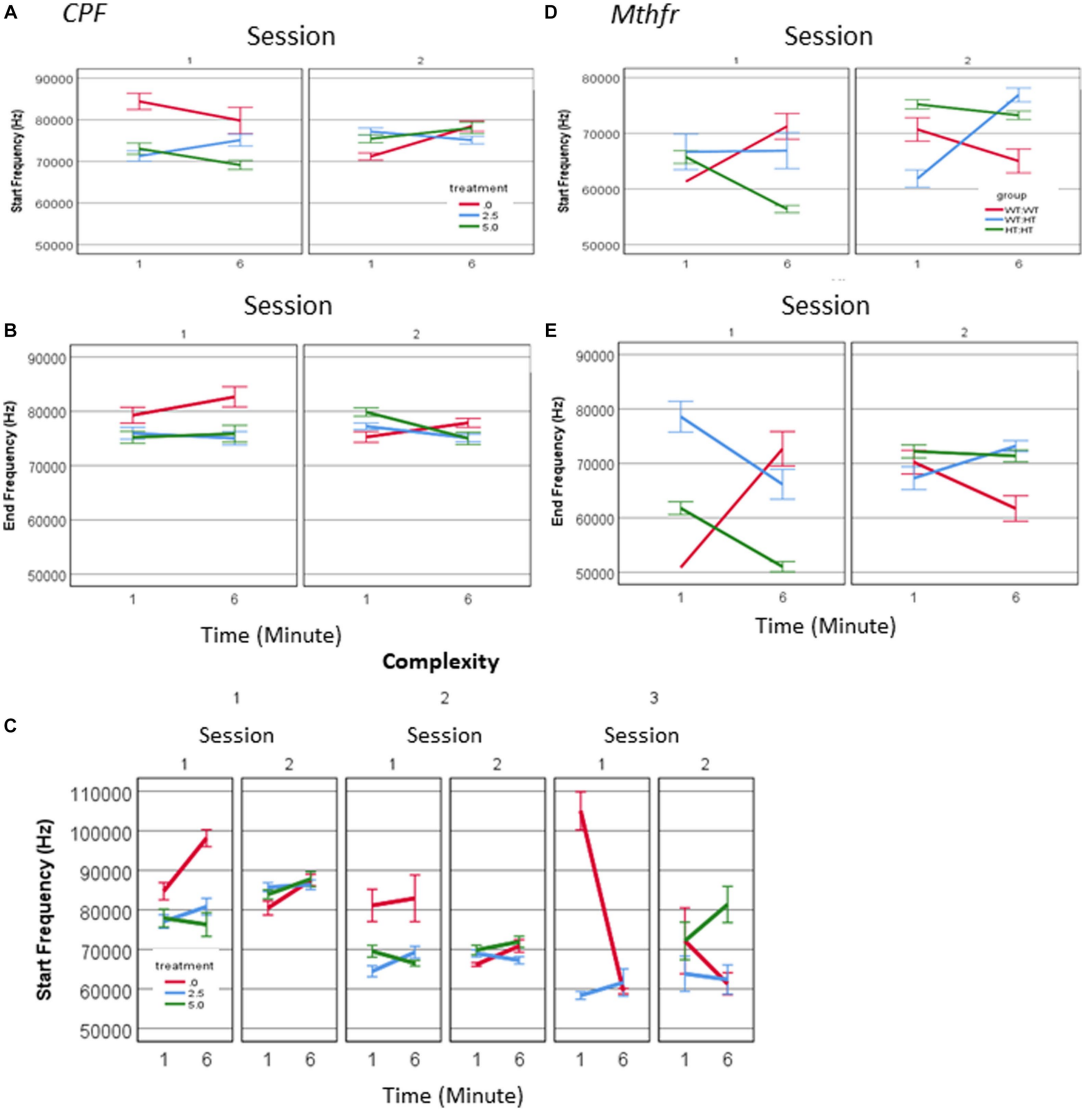
CPF treatment and Mthfr+/− genotype alters the spectral properties of male pup isolation induced vocalization. The effect of CPF on the mean frequency of USVs at the start and end of the call **(A,B)** and the mean frequency at the start of the calls presented by complexity level **(C)**. The effect of Mthfr+/− genotype on the mean frequency of USVs at the start and end of the call **(D,E)**. Mean +/− SE, N = CPF: 2,113 (Oil: 463, CPF-L: 995, CPF-H-655), Mthfr—925 (Wt:Wt-157, Het:Wt-212, Het:Het-560).

There was a three-way interaction between treatment * session * minute (*F*_2,1,594_ = 11.051, *p* = 0.000), for the frequency at the start of the call, as shown in Figure 5. In the first session a significant effect of treatment was observed (*F*_2,1,594_ = 17.485, *p* = 0.000) and there was an interaction between treatment and minute (*F*_2,1,594_ = 4.824, *p* = 0.000), where the start frequency in the CPF groups was 84% and 87% of control (CPF-L and CPF-H, respectively), in the 1st minute and see Figures 5A,B and Supplementary Table 2. In S2, we also observed an interaction between the treatment * minute (*F*_2,1,502_ = 10.380, *p* = 0.000), with a reversal of the relationship among the groups. The average start frequency of the Vehicle group in S2 was 4,000–5,000 Hz lower than it was in S1, whereas the CPF groups showed increased start frequency to 127 and 124% of the vehicle group (CPF-L and CPF-H, respectively). Frequency at the end of the call interacted with treatment * session (*F* = 6.114, *p* = 0.002). In S1 CPF treated pups showed a lower end frequency to 91%–96% of the control (see Supplementary Table 2), and in S2, an interaction between treatment and minute (*F*_2,1,502_ = 8.601, *p* = 0.000) manifested itself mainly by a higher end call frequency in the 1st min and a lower frequency in the 6th min compared to Vehicle (Supplementary Table 2). This again is characterized by a reversal in the relationship among the groups as a consequence of the treatment. In S1, the end frequency was higher in the Vehicle group compared to CPF groups, but in S2 there was no significant difference among the groups. In summary, the vehicle treated group reduced both the start and the end frequency between sessions, whereas the CPF treated groups remained stable with respect to USV frequency.

These differences were observed for calls at all levels of complexity. When analyzing calls with a single vowel (complexity level 1) we observed in the vehicle group higher frequencies in S1, compared to S2 (an effect of sessions, *F*_1,192_ = 10.48, *p* = 0.001, *F*_1,192_ = 11.96, *p* = 0.001, start and end frequency, respectively) and higher frequencies in the 1st compared to the 6th min of each session (effect of minutes of the test, *F*_1,192_ = 19.82, *p* = 0.000, *F*_1,192_ = 7.93, *p* = 0.005, start and end respectively; Figure 5C). An effect of session on Level 1 call frequencies was observed in the CPF-H and CPF-L groups, but not the time during each session. Spectral properties of calls with higher complexity order (Level 2) in the vehicle group present higher frequencies at S1 compared to S2 (effects of session, *F*_1,131_ = 31.378, *p* = 0.000 and *F*_1,131_ = 9.569, *p* = 0.000, start and end frequency, respectively), with no effect of minute (time during the session). The absence of the effect of time was also observed in the groups exposed *in-utero* to CPF.

In male pups of the Mthfr model, interactions between the effects of genotype * session (*F*_1,847_ = 16.24, *p* = 0.000), and genotype * minute (*F*_1,847_ = 57.742, *p* = 0.000) were found for the frequency at the start of the call, with the higher frequencies in the 6th minute of S1 and S2. An interaction between genotype and session was observed also for the end frequency (*F*_1,847_ = 33.003, *p* < 0.0001, and effect of genotype *F*_1,847_ = 22.299, *p* < 0.0001). Thus, both start and end frequencies of pups’ calls show time-dependent sensitivity to offspring genotype (Figures 5D,E). The effect of maternal genotype on spectral variables was more complex, presenting an interaction between maternal genotype * session * min [*F*_1,847_ = 5.244, *p* = 0.022 (start frequency), *F*_1,847_ = 9.861, *p* = 0.002 (end frequency)].

### The effect of sex on USVs production by pups of the Mthfr ASD model

The number of male pups emitting USV calls increased following reunion with the litter (S2), while female pups of all groups emitted calls upon first isolation session (Supplementary Table 3).

**Figure 6:**
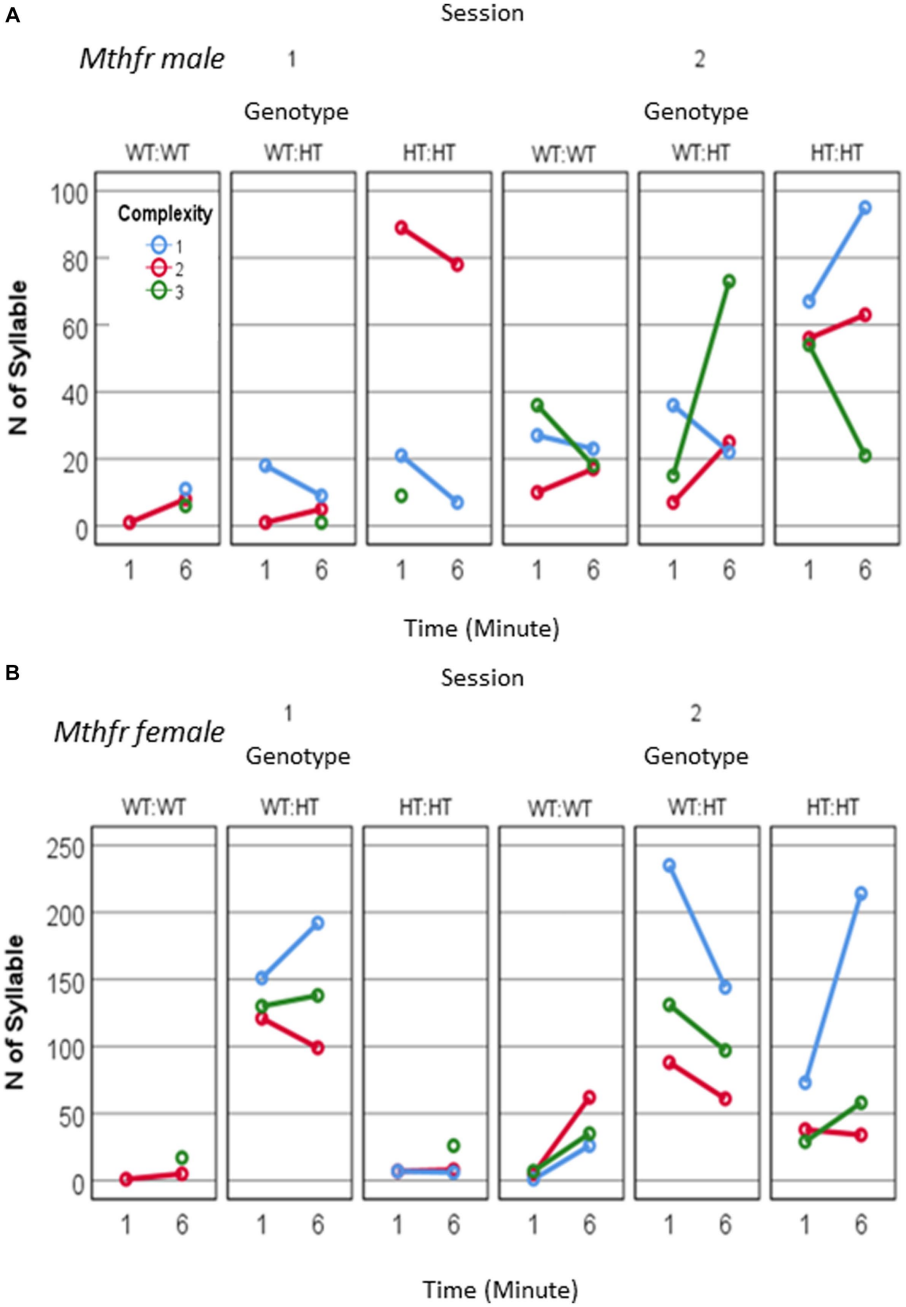
The effect of pup sex and Mthfr+/− genotype on isolation induced USV calls quantity and complexity. Mthfr genotype alters the number and complexity of isolation induced USV calls emitted by male and female pups, in a sex and time dependent manner. **(A)** Male, **(B)** Female. Mean +/− SE.

Potentiation in the number of calls/pup was observed in the females (effect of session, *F*_11, 63_ = 4.374, *p* = 0.041) which show also an interaction between session and genotype (*F*_11, 63_ = 4.743, *p* = 0.034). The increase in the number of calls in S2 was observed in the Wt-Wt and Het-Het groups but not in Het-Wt group. In the males potentiation in the number of calls/pup shows a trend for the effect of session as reported above (effect of session *F*_11, 35_ = 3.741, *p* = 0.065) as shown in Figures 6A,B and Supplementary Table 3. Thus, an interaction between maternal and offspring Mthfr genotype affected the reunion (mother and litter) induced an increase in the number of pups’ calls.

### The effect of Mthfr genotype and sex on call properties

**Figure 7:**
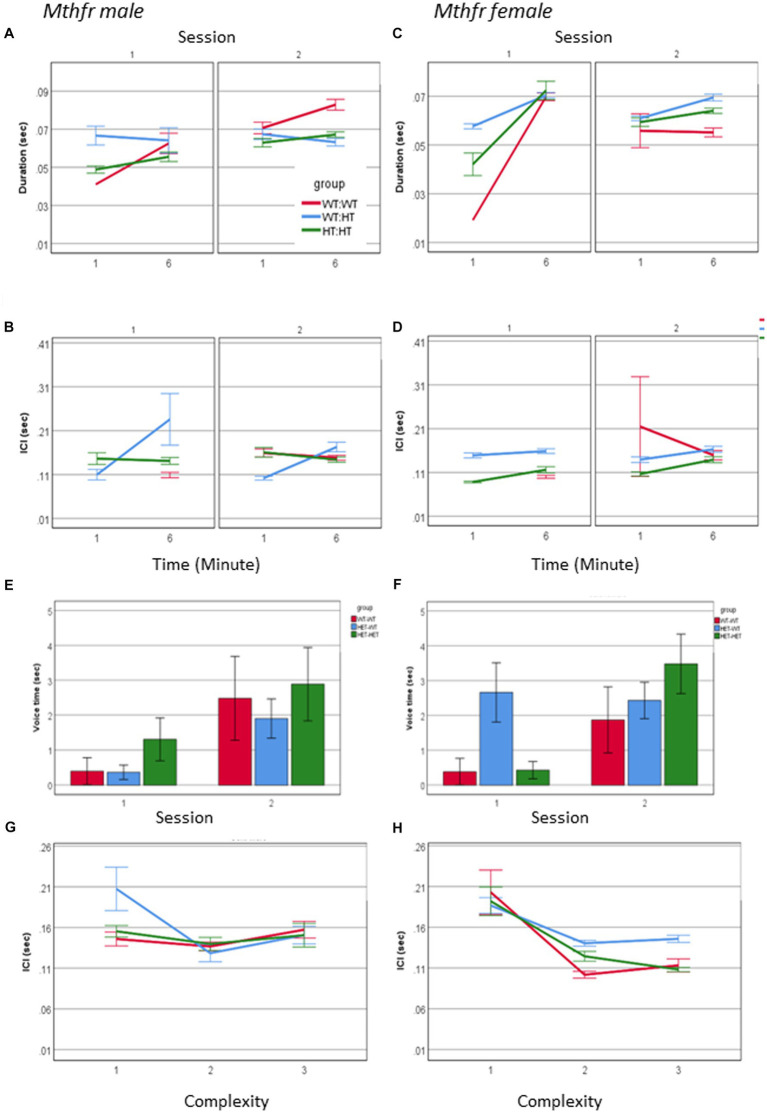
The effect of pup sex and Mthfr+/− genotype on the temporal properties of isolation induced USV calls. Duration **(A,B)**, ICI **(C,D)**. Mean +/− SE, N = Male: 925 (Wt:Wt-157, Het:Wt-212, Het-Het-560), Female: 2,246 (Wt:Wt-159, Het:Wt-1587, Het:Het-500). Voice time **(E,F)**, and the relations between ICI and call complexity **(G,H)**. Mean +/− SE, N = Male: 9 (Wt:Wt-2, Het:Wt-3, Het:Het-4), Female: 16 (Wt-Wt-2, Het-Wt-10, Het-Het-4).

An interaction between maternal Mthfr genotype * offspring Mthfr genotype * session * minute * sex was observed for call duration and ICI (*F*_6, 3177_ = 3.495, *p* = 0.001 and *F*_6, 2,498_ = 5.623, *p* = 0.000, respectively). In order to understand this interaction, the effect of genotype, session and minute was analyzed separately for male and female pups. While during S1 call duration and ICI in both sexes were affected with similar tendencies, during S2 there was an interaction of these variables with sex with a stronger effect in the female pups. The shorter ICI was associated with maternal genotype, as demonstrated by the percent of change compared to the Wt-Wt group, shown in Figures 7A–D and Supplementary Table 4. Genotype affected the duration of voice time in females as shown by the interaction between offspring genotype and session *F*_1,63_ = 4.098, *p* = 0.048, with the wt offspring preserving similar voice duration in both sessions and the Mthfr+/− offspring increasing voice time during S2, compared to S1. This interaction was not present in the male pups (Figures 7E,F). Finally, female pups, unlike male pups, show a negative correlation between ICI and call complexity level (R = −0.155, *p* < 0.0001), with a minute difference between groups, as shown in Figures 7G,H.

**Figure 8:**
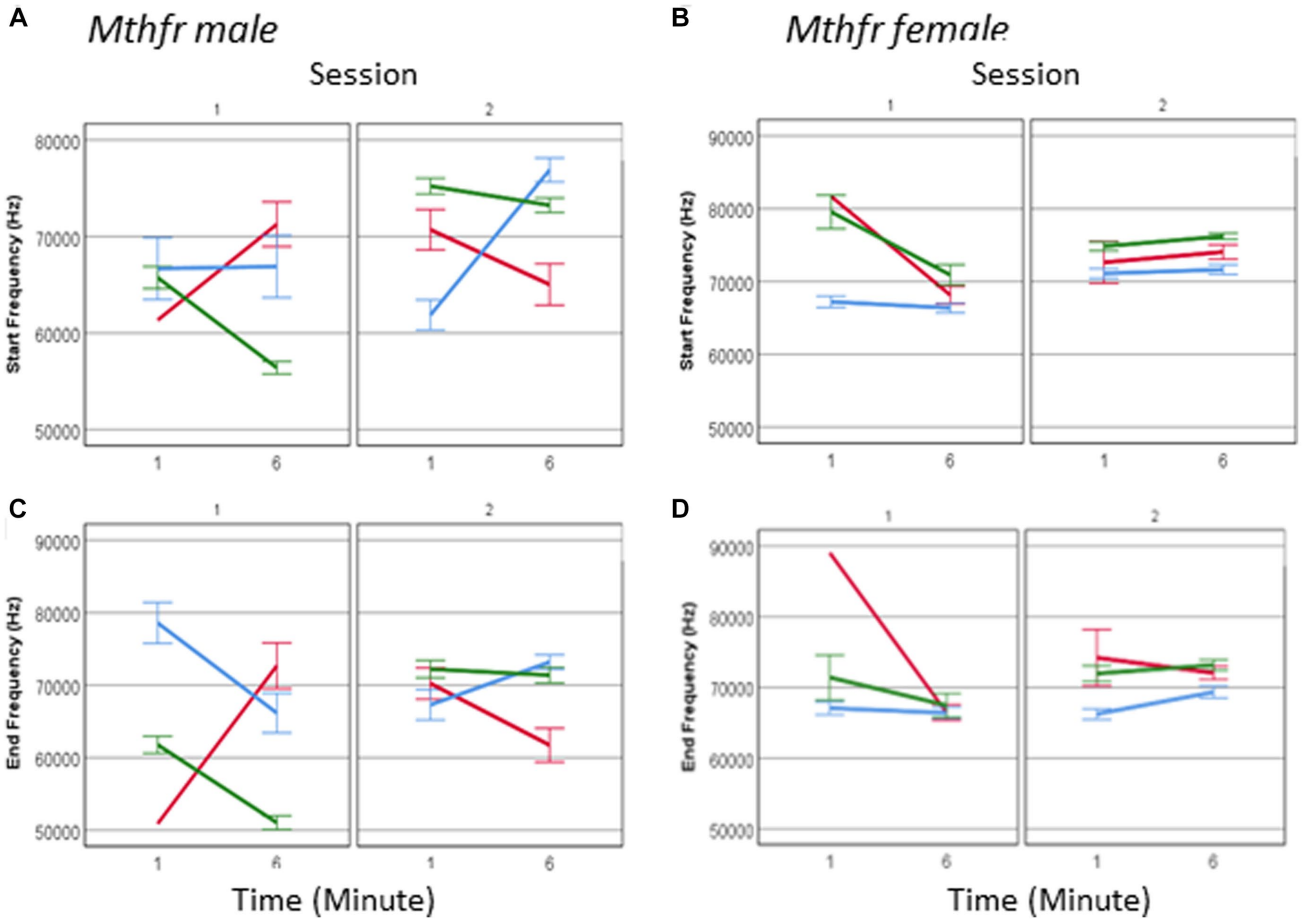
The effect of pup sex and Mthfr+/− genotype on the spectral properties of isolation induced USV calls. Mean frequency at the start of the USV call, in male and female **(A,B)** and at the end of the USV call **(C,D)**. Mean +/− SE, N = Male: 925 (Wt:Wt-157, Het:Wt-212, Het:Het-560), Female: 2,246 (Wt:Wt-159, Het:Wt-1,587, Het:Het-500).

The spectral properties of pup calls, expressed by the start and end frequencies, show an interaction between offspring Mthfr genotype * session * sex (*F*_6, 2,975_ = 19.835, *p* = 0.001 and *F*_6, 2,975_ = 14.570, *p* = 0.001, respectively; Figures 8A–D). As in the males, in female pups offspring genotype affected start and end frequency of USVs calls (*F*_df1,2,128_ = 35.714, *p* < 0.0001 and *F*_1,2,128_ = 22.299, *p* < 0.0001, respectively), with the pups of the Wt-Het group presenting the lowest frequencies during the whole test. The interaction between offspring genotype and sex reveals that the female Mthfr+/− pups have higher frequencies compared to the Wt, while in the males this is not the case, as shown in Figure 8. The overall effect of pup sex on call start and end frequency (*F*_6,2,975_ = 10.573, *p* = 0.001 and *F*_6,2,975_ = 8.497, *p* = 0.004, respectively), is illustrated also in Supplementary Table 4, showing that maternal Mthfr-deficiency is associated with an increase in start and end frequency at the beginning of S1, in male pups, whereas in the females the opposite effect is observed. In addition, in the males an effect of Mthfr genotype is maintained at later times as described above, while in the female pups, no effect of Mthfr genotype was observed at the later time point (Minute 6).

One may suggest that the lack of effect in female pups is restricted to a specific call type. To examine this possibility, we analyzed female call frequency for the effect of Mthfr genotype at calls at each complexity level. Indeed, a significant effect of offspring Mthfr+/− genotype (*F*_8,434_ = 26.447, *p* = 0.000 and *F*_8,434_ = 14.785, *p* = 0.000, start and end, respectively) and of maternal Mthfr+/− genotype (*F*_8,434_ = 5.062, *p* = 0.025 start frequency) was found on level 1 calls and level 3 calls (offspring Mthfr genotype, *F*_8,610_ = 18.373, *p* = 0.000 and *F*_8,610_ = 20.391, *p* = 0.000, start and end, respectively and maternal Mthfr genotype *F*_8,610_ = 13.318, *p* = 0.000 and *F*_8,610_ = 21.457, *p* = 0.000, start and end, respectively). Due to higher variability in the frequency steps of calls with complexity level 2, the spectral features show high variability and the effect of Mthfr genotype is not statistically significant.

## Discussion

Dynamic alteration of isolation induced calls was observed following gestational exposure to chlorpyrifos, a organophosphate pesticide that was widely used, but recently banned in the USA and Europe, and in the genetic model of autism related to folate metabolism. The number of calls, temporal pattern of call usage, call duration, rate, and spectral properties of the calls in male pups of both models of ASD deviated from their control groups. Most of these variables dynamically changed throughout the experiment (minutes within a session and between sessions). A feature of pup behavior that was preserved in all male pup groups (with a varying level) was the enhancement in pups’ reaction to the second isolation session, which is called maternal potentiation. Potentiation was reflected by larger number of calling pups, the number of calls emitted by each pup, and voice duration, in the second isolation session, following the reunion with the dam and littermates, compared to the response to the first isolation session. Maternal potentiation of pup response requires learning the reinforcing effect of reunion (first isolation session) on USV production, so that production will be enhanced in the subsequent isolation event. This form of learning, essential for pup survival, was not impaired in male pups in both models of ASD-like behavior.

In addition to the response to reunion with the dam, we report here an effect of time within the sessions on the number of calls and all measured features. The dynamic nature of call features may depend on physiological factors such as fatigue and emotional factors such as stress. Regulation of USV call emission, is tightly related to pup breathing (Portfors, 2007; Wei et al., 2022) and its “arousal state” (Rieger and Dougherty, 2016; Pranic et al., 2022). It was suggested that the temporal variables such as duration and ICI are associated with motor control by central generators of breathing rhythm, while the spectral properties of the calls are dictated by the laryngeal structures with several laryngeal elements involved in voice production and its spectral properties (such as the ventral pouch; Riede et al., 2017, 2020; Abhirami et al., 2023). The structural elements undergo extensive growth during early postnatal days of mice pup life and contribute to the developmental changes in the spectral properties of USV calls (Riede et al., 2020). Although USVs production depends on air flow during breathing, the number of calls emitted is not linearly related to respiratory parameters, as shown in the glycine transporter 2 deficient mice (Hülsmann et al., 2019). These pups present severe impairment of respiratory parameters without an effect on the acoustic structure of the calls. In addition, they emitted more USVs than pups from the control group.

Considering CPF is an organo-phosphate chemical, it is possible to suspect that prenatal exposure to CPF interferes with the growth or morphology of the larynx or some of its elements. However, following reunion with the mother, call features did not differ from the vehicle group, excluding the possibility of physical/structural difference between the groups. It is more plausible that gestational CPF modifies the neuronal circuit/s activating voice production, including regulation of central patterning generators and larynx activation. The larynx is controlled by the vagus nerve, providing a plausible route for modification of USVs by stress and activity. In another model of anomalous development following gestational exposure to a toxin, *in utero* dioxin exposure on GND 12.5 in mice led to shorter duration USV only when body temperature was lower than 37°C and also to fewer complex calls in P3 pups (Kimura and Tohyama, 2018). In contrast to the CPF and MTHFR models, the pups in this developmental model had no postnatal sensorimotor deficits and no abnormalities of vocal fold structure. Arousal state and stress are possible modulators of pup response (Pranic et al., 2022).

Another feature of vocalization affected by mechanical and emotional aspects is the call complexity. A deviation between simple calls with a single main frequency, more complex calls containing more than one frequency jumps and harmonic calls was recently used in several studies (Agarwalla et al., 2020; Nolan et al., 2020; Yang et al., 2021). For example, head restraint decreased the use of simple calls and increased the use of USV with syllable jumps (similar to complexity level 2), which may indicate association with stress (Weiner et al., 2016). Decreased use of simple calls and increased use of harmonic USVs in pups was observed following neonatal seizures (P10) in FMR1-KO mice (Huebschman et al., 2020). Analyzing the calls by their complexity in the current study, revealed an increased emission of harmonic calls (complexity level 3), both in CPF-Hi pups and offspring to Mthfr+/− dams. Moreover, in the experimental groups of both models tested here, the emission of harmonic calls was enhanced following reunion with the nest. Since this type of call was barely used at the first isolation session, the increased use of harmonic calls suggests a response to higher distress. Harmonic calls, which are also longer, contribute to prolonging voice time in the second isolation session. It is not known if dams respond more readily to level 3 calls, but this issue is worth exploring given the finding that these calls were more prevalent in S2.

Pup post-reunion behavior and vocalization is affected by the encounter with the dam, mediated by the quality of physical maternal care and synchronization of vocal communication between pup and dam during the reunion session (Guoynes and Marler, 2021). In accordance with this observation, impaired maternal care induced by exposure to the sedative drug clozapine, influenced post-reunion vocalization in rat pups (Li et al., 2011). As evident in previous studies, the changes in pup vocalization following reunion with the dam depend on maternal behavior during reunion, and therefore can be regarded as modulation of neuronal circuits regulating state and not to structural differences.

Last, as evident in other models of ASD FMR1 (Reynolds et al., 2016), PTEN (Binder and Lugo, 2017), CBD (Iezzi et al., 2022), and 16p11.2 deletion (Agarwalla et al., 2020), pups sex affected different aspects of USVs emission in the Mthfr model. In contrast to the male pups, female pups in the Mthfr model and its control group emitted calls at both sessions of recording. Potentiation of the number of calls emitted following reunion was not observed in the Wt offspring of Mthfr+/− dams. These pups emitted similar numbers of calls and similar voice time before and after reunion with the nest and dam. USV calls features both in male and female of the Mthfr model were sensitive to offspring genotype, with deviation in the temporal dynamics of changes within and between the isolation sessions. A limitation of this paper is that we did not have sufficient mice to analyze the USVs of females exposed to gestational chlorpyrifos.

Altogether, examining the time course of pups USV communication during two consecutive isolation sessions, demonstrated that in both models of ASD, USV quantity and acoustic structure changes dynamically within each session and between the sessions, with the first isolation session was characterized by a buildup of call quantity and significant effect on USV acoustic structure, whereas the second isolation session was characterized by enhanced calls and voice time, with minimal modification of USV spectral features. The finding of such temporal dynamics of calls should be considered in future USV communication analysis in rodent models of ASD and other psychiatric disorders to maximize the sensitivity of the study. Furthermore, temporal exploration of sets of data available from other ASD models may provide generalization of this observation.

## Data availability statement

The raw data supporting the conclusions of this article will be made available by the authors, without undue reservation.

## Ethics statement

The animal study was approved by the Animal Care and Use Committee of Ben-Gurion University of the Negev, Israel (protocols IL-16-07-14 and IL-66-11-13). The study was conducted in accordance with the local legislation and institutional requirements.

## Author contributions

AG: Methodology, Data curation, Formal analysis, Writing – review & editing. ER: Data curation, Methodology, Writing – review & editing. SG: Data curation, Methodology, Writing – review & editing. DL: Methodology, Investigation, Software, Validation, Writing – review & editing. OK: Investigation, Methodology, Validation, Conceptualization, Funding acquisition, Supervision, Writing – original draft. HG: Conceptualization, Funding acquisition, Investigation, Methodology, Supervision, Validation, Writing – original draft.
