# Supplementary material for: Temporal dynamics of isolation calls emitted by pups in environmental and genetic mouse models of autism spectrum disorder
Source: Front Neurosci. 2023 Oct 23;17:1274039. doi: 10.3389/fnins.2023.1274039 (PMC10629105; doi:10.3389/fnins.2023.1274039)
Supplement: Supplementary file 1 [file Data_Sheet_1.PDF]

Supplementary Table 1-The number of calls emitted by CPF and Mthfr male pups.

Session	Minute	CPF mg/Kg	MG	OG	Calls (N)	Calls Lvl 1	Calls Lvl 2	Calls Lvl 3	Calls/Pup (all)	Calls/Pup SD	Call/pup (Vocal)	Call /pup (vocal) SD	Pups (N)	Vocal pups (N)	Vocal Pups %	Calls /pup % change*	Vocal pups % change**	Calls/pup % change**	Vocal pups % change**
1	1	oil			59	40	17	2	8.429	15.576	14.750	15.58	7	4	57.14				
1	1	2.5			170	92	74	4	17.000	24.272	24.286	24.27	10	7	70.00	201.69	122.5		
1	1	5			117	50	67	0	13.000	23.948	23.400	23.95	9	5	55.56	154.24	97.22		
1	6	oil			45	17	13	15	6.429	11.900	11.250	11.90	7	4	57.14				
1	6	2.5			116	61	51	4	11.600	15.145	16.571	15.15	10	7	70.00	180.44	122.5		
1	6	5			70	19	51	0	7.778	18.640	17.500	18.64	9	4	44.44	120.99	77.77		
2	1	oil			181	62	114	5	25.857	29.947	30.167	29.95	7	6	85.71			306.78	150.00
2	1	2.5			369	182	179	8	36.900	32.865	46.125	32.86	10	8	80.00	142.71	93.33	217.06	114.29
2	1	5			296	114	158	24	32.889	25.394	32.889	25.39	9	9	100.00	127.19	116.66	252.99	180.00
2	6	oil			178	83	90	5	25.429	27.646	25.429	27.65	7	7	100.00			395.56	175.00
2	6	2.5			340	146	183	11	34.000	33.876	37.778	33.88	10	9	90.00	133.71	90	293.10	128.57
2	6	5			172	47	91	34	19.111	14.920	21.500	14.92	9	8	88.89	75.16	88.88	245.71	200.00
1	1		WT	WT	1	1	0	0	0.500	0.707	1.000	0.71	2	1	50.00				
1	1		HT	WT	19	1	18	0	6.333	10.970	19.000	10.97	3	1	33.33	1266.67	66.66		
1	1		HT	HT	119	89	21	9	29.750	34.942	39.667	34.94	4	3	75.00	5950.00	150		
1	6		WT	WT	25	8	11	6	12.500	17.678	25.000	17.68	2	1	50.00				
1	6		HT	WT	15	5	9	1	5.000	5.000	7.500	5.00	3	2	66.67	40.00	133.33		
1	6		HT	HT	85	78	7	0	21.250	37.977	42.500	37.98	4	2	50.00	170.00	100		
2	1		WT	WT	73	10	27	36	36.500	50.205	36.500	50.20	2	2	100.00			7300.00	200.00
2	1		HT	WT	58	7	36	15	19.333	25.929	19.333	25.93	3	3	100.00	52.97	100	305.26	300.00
2	1		HT	HT	177	56	67	54	44.250	50.737	44.250	50.74	4	4	100.00	121.23	100	148.74	133.33
2	6		WT	WT	58	17	23	18	29.000	22.627	29.000	22.63	2	2	100.00			232.00	200.00
2	6		HT	WT	120	25	22	73	40.000	7.550	40.000	7.55	3	3	100.00	137.93	100	800.00	150.00
2	6		HT	HT	179	63	95	21	44.750	38.405	44.750	38.40	4	4	100.00	154.31	100	210.59	200.00

The number of pups and calls emitted in each of the minutes/sessions, and the percent of change. Maternal genotype – MG, Offspring genotype – OG, The number of calls of complexity level 1 -Lvl1 (same for Lvl2 and Lvl3). Pups that emitted at least 1 USV during the relevant minute - Vocal pups. Percent of change compared to control = *, Percent of change compared to the parallel minute in S1 = **. Red- percent of change is 5% or more higher, Blue – percent of change is 5% or more lower than the compared value.

Supplementary Table 2- **Spectral and temporal features of USV calls emitted by CPF and Mthfr male pups.**

Session	Minute	CPF mg/Kg	MG	OG	Pup (N)	Calls (N)	Valid Calls(N)	Duration [ms]	Duration (ms) SD	ICI [ms]	ICI [ms] SD	Start Frequency [Hz]	Start Frequency [Hz] SD	End Frequency [Hz]	End Frequency [Hz] SD	Duration %	ICI %	Start Frequency %	End Frequency %
1	1	oil			7	59	57	0.036	0.017	0.183	0.101	84421	14702	79269	11062				
1	1	2.5			10	170	166	0.043	0.035	0.212	0.143	71288	15781	75960	14142	118%	116%	84%	96%
1	1	5			9	117	116	0.049	0.030	0.227	0.139	73068	14438	75208	11772	135%	124%	87%	95%
1	6	oil			7	45	40	0.046	0.024	0.157	0.080	79823	19975	82659	11810				
1	6	2.5			10	116	115	0.047	0.027	0.187	0.113	75116	15279	75031	13197	102%	119%	94%	91%
1	6	5			9	70	70	0.061	0.030	0.292	0.116	69143	9066	75872	12961	131%	186%	87%	92%
2	1	oil			7	181	180	0.051	0.032	0.177	0.067	71179	11482	75257	12979				
2	1	2.5			10	369	361	0.046	0.027	0.185	0.095	77187	16171	77234	11467	91%	105%	108%	103%
2	1	5			9	296	290	0.042	0.024	0.163	0.081	75451	16177	79862	13031	84%	92%	106%	106%
2	6	oil			7	178	173	0.040	0.022	0.210	0.087	78471	16372	77869	10791				
2	6	2.5			10	340	333	0.051	0.028	0.203	0.115	75120	16456	75081	12832	127%	96%	96%	96%
2	6	5			9	172	165	0.050	0.022	0.203	0.113	78040	17749	75002	14732	124%	96%	99%	96%
1	1		WT	WT	2	1	1	0.041	0.000			61338	0	50884	0				
1	1		HT	WT	3	19	19	0.067	0.021	0.110	0.048	66670	13960	78585	12322	162%		109%	154%
1	1		HT	HT	4	119	118	0.049	0.020	0.146	0.126	65737	12464	61782	12817	119%		107%	121%
1	6		WT	WT	2	25	25	0.063	0.027	0.109	0.029	71256	11552	72682	15791				
1	6		HT	WT	3	15	13	0.064	0.024	0.236	0.186	66884	11603	66167	9875	103%	217%	94%	91%
1	6		HT	HT	4	85	77	0.056	0.022	0.141	0.063	56398	5725	51029	8265	89%	130%	79%	70%
2	1		WT	WT	2	73	73	0.071	0.026	0.159	0.073	70698	17865	70233	18482				
2	1		HT	WT	3	58	58	0.067	0.021	0.102	0.031	61870	11868	67291	15950	95%	64%	88%	96%
2	1		HT	HT	4	177	156	0.063	0.028	0.161	0.125	75209	10243	72217	14969	89%	101%	106%	103%
2	6		WT	WT	2	58	58	0.083	0.022	0.148	0.041	65026	16327	61729	17757				
2	6		HT	WT	3	120	102	0.063	0.020	0.173	0.100	76891	12562	73187	10052	76%	117%	118%	119%
2	6		HT	HT	4	179	159	0.067	0.018	0.144	0.071	73235	9473	71382	13565	81%	97%	113%	116%

The number of pups and calls emitted in each of the minutes/sessions. The number of calls refers to the total number of calls emitted by the pups that have information regarding their call type and complexity level. Valid calls are those that have a full set of data including type, complexity and all spectral and temporal information. Maternal genotype – MG, Offspring genotype – OG, inter call interval – ICI. Percent of change was calculated compared to control. Red- percent of change is 5% or more higher, Blue – percent of change is 5% or more lower than the compared value.

Supplementary Table 3- The number of calls emitted by male and female pups of the Mthfr model of ASD.

Session	Minute	Sex	MG	OG	Calls (N)	Calls Lvl 1	Calls Lvl 2	Calls Lvl 3	Calls/Pup	Calls/Pup (SD)	Pups Count	Vocal Pups (N)	Vocal Pups (%)	calls/pup % difference *	Vocal pups %difference*	calls/ pups % change**	Vocal pups %change**
1	1	male	WT	WT	1	1	0	0	0.5	0.7	2	1	50.0				
1	1	male	HT	WT	19	1	18	0	6.3	11.0	3	1	33.3	1266.67	66.66		
1	1	male	HT	HT	119	89	21	9	29.8	34.9	4	3	75.0	5950.00	150		
1	6	male	WT	WT	25	8	11	6	12.5	17.7	2	1	50.0				
1	6	male	HT	WT	15	5	9	1	5.0	5.0	3	2	66.7	40.00	133.33		
1	6	male	HT	HT	85	78	7	0	21.3	38.0	4	2	50.0	170.00	100		
2	1	male	WT	WT	73	10	27	36	36.5	50.2	2	2	100.0			7300	200
2	1	male	HT	WT	58	7	36	15	19.3	25.9	3	3	100.0	52.97	100	305.26	300
2	1	male	HT	HT	177	56	67	54	44.3	50.7	4	4	100.0	121.23	100	148.73	133.3333
2	6	male	WT	WT	58	17	23	18	29.0	22.6	2	2	100.0			232	200
2	6	male	HT	WT	120	25	22	73	40.0	7.5	3	3	100.0	137.93	100	800	150
2	6	male	HT	HT	179	63	95	21	44.8	38.4	4	4	100.0	154.31	100	210.52	200
1	1	female	WT	WT	1	1	0	0	0.5	0.7	2	2	100.0				
1	1	female	HT	WT	402	121	151	130	40.2	63.9	10	10	100.0	8040.00	100		
1	1	female	HT	HT	14	7	7	0	3.5	3.1	4	4	100.0	700.00	100		
1	6	female	WT	WT	22	5	0	17	11.0	15.6	2	2	100.0				
1	6	female	HT	WT	429	99	192	138	42.9	54.8	10	10	100.0	390.00	100		
1	6	female	HT	HT	40	8	6	26	10.0	11.5	4	4	100.0	90.91	100		
2	1	female	WT	WT	13	5	1	7	6.5	2.1	2	2	100.0			1300	100
2	1	female	HT	WT	454	88	235	131	45.4	31.3	10	10	100.0	698.46	100	112.93	100
2	1	female	HT	HT	140	38	73	29	35.0	13.8	4	4	100.0	538.46	100	1000	100
2	6	female	WT	WT	123	62	26	35	61.5	2.1	2	2	100.0			559.09	100
2	6	female	HT	WT	302	61	144	97	30.2	29.5	10	10	100.0	49.11	100	70.396	100
2	6	female	HT	HT	306	34	214	58	76.5	42.8	4	4	100.0	124.39	100	765	100

The number of pups and calls emitted in each of the minutes/sessions, and the percent of change. Maternal genotype – MG, Offspring genotype – OG. The number of calls of complexity level 1 -Lvl1 (same for Lvl2 and Lvl3). Pups that emitted at least 1 USV during the relevant minute - Vocal pups. Percent of change compared to control (WT:WT) of the same sex = *, Percent of change compared to the parallel minute in S1 = **. Red- percent of change is 5% or more higher, Blue – percent of change is 5% or more lower than the compared value.

Supplementary Table 4 - Spectral and temporal features of USV calls emitted male and female pups of the Mthfr model of ASD.

Session	Minute	Sex	MG	OG	Pup (N)	Calls (N)	Valid Calls (N)	Duration [ms]	Duration (ms) SD	ICI [ms]	ICI [ms] SD	Start Frequency [Hz]	Start Frequency [Hz] SD	End Frequency [Hz]	End Frequency [Hz] SD	Duration %	ICI %	Start Frequency %	End Frequency %
1	*1*	*Male*	*WT*	*WT*	*2*	*1*	*1*	*0.041*	0.000			*61338*	0	*50884*	0				
1	1	Male	HT	WT	3	19	19	0.067	0.021	0.110	0.048	66670	13960	78585	12322	162%		109%	154%
1	1	Male	HT	HT	4	119	118	0.049	0.020	0.146	0.126	65737	12464	61782	12817	119%		107%	121%
1	*6*	*Male*	*WT*	*WT*	*2*	*25*	*25*	*0.063*	0.027	*0.109*	0.029	*71256*	11552	*72682*	15791				
1	6	Male	HT	WT	3	15	13	0.064	0.024	0.236	0.186	66884	11603	66167	9875	103%	217%	94%	91%
1	6	Male	HT	HT	4	85	77	0.056	0.022	0.141	0.063	56398	5725	51029	8265	89%	130%	79%	70%
2	*1*	*Male*	*WT*	*WT*	*2*	*73*	*73*	*0.071*	0.026	*0.159*	0.073	*70698*	17865	*70233*	18482				
2	1	Male	HT	WT	3	58	58	0.067	0.021	0.102	0.031	61870	11868	67291	15950	95%	64%	88%	96%
2	1	Male	HT	HT	4	177	156	0.063	0.028	0.161	0.125	75209	10243	72217	14969	89%	101%	106%	103%
2	*6*	*Male*	*WT*	*WT*	*2*	*58*	*58*	*0.083*	0.022	*0.148*	0.041	*65026*	16327	*61729*	17757				
2	6	Male	HT	WT	3	120	102	0.063	0.020	0.173	0.100	76891	12562	73187	10052	76%	117%	118%	119%
2	6	Male	HT	HT	4	179	159	0.067	0.018	0.144	0.071	73235	9473	71382	13565	81%	97%	113%	116%
1	*1*	*female*	*WT*	*WT*	*2*	*1*	*1*	*0.019*	0.000			*81687*	0	*89003*	0				
1	1	female	HT	WT	10	402	382	0.058	0.020	0.149	0.101	67211	15243	67069	17754	301%		82%	75%
1	1	female	HT	HT	4	14	14	0.042	0.017	0.088	0.004	79561	8625	71398	11888	220%		97%	80%
1	*6*	*female*	*WT*	*WT*	*2*	*22*	*22*	*0.070*	0.007	*0.100*	0.014	*68133*	5667	*66444*	5267				
1	6	female	HT	WT	10	429	404	0.070	0.022	0.159	0.100	66355	13158	66385	15400	101%	159%	97%	100%
1	6	female	HT	HT	4	40	39	0.072	0.024	0.116	0.040	70904	8679	67460	10519	104%	116%	104%	102%
2	*1*	*female*	*WT*	*WT*	*2*	*13*	*13*	*0.056*	0.025	*0.215*	0.360	*72619*	10358	*74180*	14425				
2	1	female	HT	WT	10	454	428	0.061	0.020	0.139	0.120	71097	13918	66229	15477	109%	65%	98%	89%
2	1	female	HT	HT	4	140	138	0.059	0.021	0.106	0.048	74819	7053	71951	13259	106%	50%	103%	97%
2	*6*	*female*	*WT*	*WT*	*2*	*123*	*123*	*0.055*	0.020	*0.149*	0.106	*74052*	11013	*72027*	10512				
2	6	female	HT	WT	10	302	280	0.069	0.023	0.163	0.107	71637	11087	69331	13882	126%	109%	97%	96%
2	6	female	HT	HT	4	306	296	0.064	0.019	0.139	0.109	76204	7312	73150	12657	116%	93%	103%	102%

The number of pups and calls emitted in each of the minutes/sessions. The number of calls refers to the total number of calls emitted by the pups that have information regarding their call type and complexity level. Valid calls are those that have a full set of data including type, complexity and all spectral and temporal information. Maternal genotype – MG, Offspring genotype – OG, inter call interval – ICI. Percent of change was calculated compared to control (WT-WT) of the same sex. Red- percent of change is 5% or more higher, Blue – percent of change is 5% or more lower than the compared value.
