# Supplementary material for: Temporal dynamics of isolation calls emitted by pups in environmental and genetic mouse models of autism spectrum disorder
Source: Front Neurosci. 2023 Oct 23;17:1274039. doi: 10.3389/fnins.2023.1274039 (PMC10629105; doi:10.3389/fnins.2023.1274039)
Supplement: Supplementary file 2 [file Data_Sheet_2.PDF]

Supplementary Material

1.1 Supplementary Figure 1

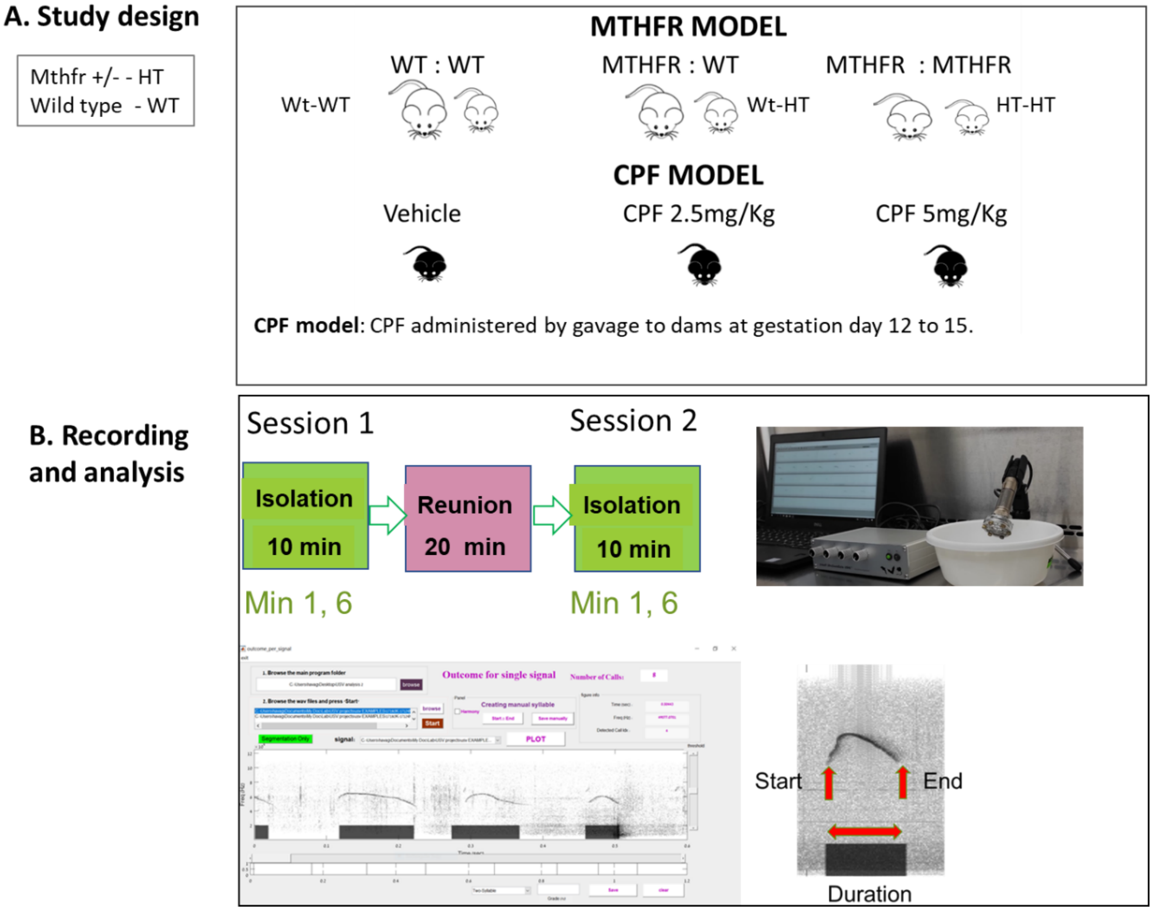

A. Study design

Mthfr mice (Balb/cAnNCrlBR background): Mthfr+/+ (wild-type [Wt]) and Mthfr+/− (heterozygote [Het]) female mice were mated with Wt males to create three groups defined by genotype and maternal genotype as follows: Wt offspring from Wt mothers (mother: offspring genotype - Wt:Wt), Wt offspring from Mthfr+/− mothers (Het:Wt) and Mthfr+/− offspring from Mthfr+/− mothers (Het:Het).

Environmental model mice (C57Bl6J (B6): Dams were divided into 3 treatment groups for organophosphate exposure (CPF) during gestation: Vehicle (VEH, corn oil), 2.5 (CPF-L) or 5 mg/kg CPF (CPF-H).

B. Recording and analysis.

Ultrasonic signals were recorded using Avisoft Bioacoustics (Berlin, Germany) system (upper right). Each pup was separated from the litter and placed in a container with no bedding to eliminate

extraneous noise. The microphone was placed 10 cm above the pup as shown in the picture. After a 10-minute isolation session (S1) the pup was placed back in the home cage with the litter for 20 minutes and separated for a second recording session of 10 minutes (S2). USV emitted during the 1st and 6th minute of each session were analyzed offline. Call variables were extracted by MATLAB based on software developed in-house shown in the lower right panel for the detection of USV calls. The time and mean frequency at the start and end of each call were extracted (left, lower panel) and USV type was classified, by a researcher blind to the group identity.

1.2 Supplementary Figure 2

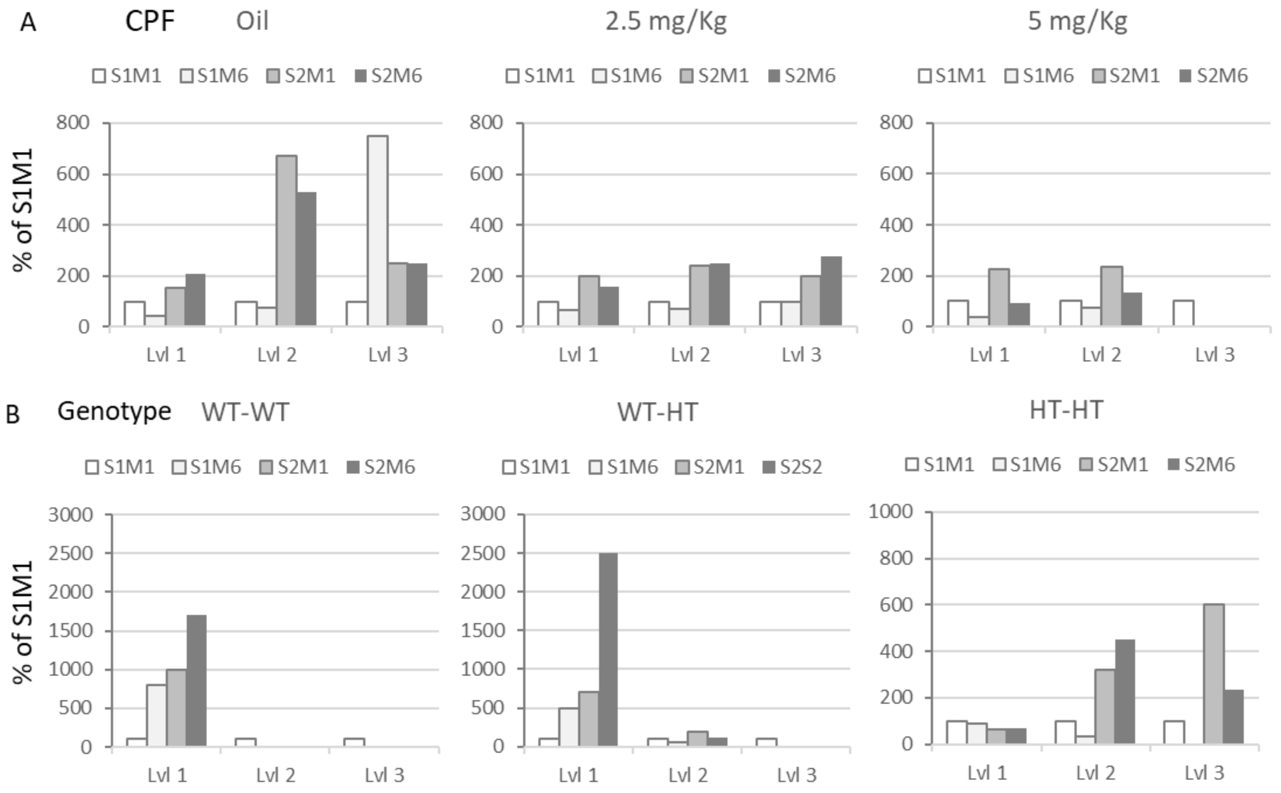

Supplementary figure 2: CPF treatment and Mthfr+/- genotype alters isolation induced call quantities and their enhancement by repeated separation from the dam. The number of USV syllables emitted by pups as a function of the number of calls emitted at the first minute of Session1 for each dosage/genetics is shown for each call complexity level. The different Y axis scales reflect the major differences between the two model strains. A. CPF model, and B. Mthfr model. Please note that when pups did not call in the first minute (Number of calls = 0), this calculation could not be done and therefore these bars are missing. Level 1, Lvl1; Level 2, Lvl2; Level 3, Lvl3, session 1 minute 1, S1M1; session 1 minute 6, S1M6; session 2 minute 1, S2M1; session 2 minute 6, S2M6.
